# The anti-aging gene *KLOTHO *is a novel target for epigenetic silencing in human cervical carcinoma

**DOI:** 10.1186/1476-4598-9-109

**Published:** 2010-05-18

**Authors:** Jaehyouk Lee, Dong-Jun Jeong, Jinsun Kim, Soonduck Lee, Jin-Hwa Park, Boogi Chang, Sam-Il Jung, Lisha Yi, Youngsoo Han, Young Yang, Keun Il Kim, Jong-Seok Lim, Inchul Yang, Seob Jeon, Dong Han Bae, Chang-Jin Kim, Myeong-Sok Lee

**Affiliations:** 1 Research Center for Women's Diseases and Division of Biological Science, Sookmyung Women's University, Seoul 140-742, Republic of Korea; 2 Department of Pathology, Soonchunhyang University College of Medicine, Cheonan 330-090, Republic of Korea; 3 Health Metrology Group, Korea Research Institute of Standards and Science, Daejeon 305-333, Republic of Korea; 4 Department of Obstetrics and Gynecology, Soonchunhyang University Cheonan Hospital, Cheonan 330-721, Republic of Korea

## Abstract

**Background:**

*Klotho *was originally characterized as an anti-aging gene that predisposed Klotho-deficient mice to a premature aging-like syndrome. Recently, KLOTHO was reported to function as a secreted Wnt antagonist and as a tumor suppressor. Epigenetic gene silencing of secreted Wnt antagonists is considered a common event in a wide range of human malignancies. Abnormal activation of the canonical Wnt pathway due to epigenetic deregulation of Wnt antagonists is thought to play a crucial role in cervical tumorigenesis. In this study, we examined epigenetic silencing of *KLOTHO *in human cervical carcinoma.

**Results:**

Loss of *KLOTHO *mRNA was observed in several cervical cancer cell lines and in invasive carcinoma samples, but not during the early, preinvasive phase of primary cervical tumorigenesis. *KLOTHO *mRNA was restored after treatment with either the DNA demethylating agent 2'-deoxy-5-azacytidine or histone deacetylase inhibitor trichostatin A. Methylation-specific PCR and bisulfite genomic sequencing analysis of the promoter region of *KLOTHO *revealed CpG hypermethylation in non-*KLOTHO*-expressing cervical cancer cell lines and in 41% (9/22) of invasive carcinoma cases. Histone deacetylation was also found to be the major epigenetic silencing mechanism for *KLOTHO *in the SiHa cell line. Ectopic expression of the secreted form of KLOTHO restored anti-Wnt signaling and anti-clonogenic activity in the CaSki cell line including decreased active β-catenin levels, suppression of T-cell factor/β-catenin target genes, such as *c-MYC *and *CCND1*, and inhibition of colony growth.

**Conclusions:**

Epigenetic silencing of *KLOTHO *may occur during the late phase of cervical tumorigenesis, and consequent functional loss of KLOTHO as the secreted Wnt antagonist may contribute to aberrant activation of the canonical Wnt pathway in cervical carcinoma.

## Background

Cervical carcinoma is the second most common cause of cancer death in women worldwide [1]. Several epidemiological studies have indicated that the human papillomavirus (HPV) provides the 'initial hit' in the development of cervical cancer [2]. It has been hypothesized that deregulation of the canonical Wnt signaling pathway is the 'second hit' in the multistep process of cervical carcinogenesis [3,4]. Aberrant activation of the Wnt/β-catenin signaling pathways has been regarded as a generic pathway in a variety of human malignancies [5,6]. Gain-of-function mutations centered in the N-terminus of *CTNNB1 *(encoding the β-catenin gene) induced these oncogenic proteins to be refractory to proteosomal degradation in many tumors. Although cytoplasmic/nuclear accumulation of β-catenin is frequently found in the malignant uterine cervix, this increase in β-catenin is not associated with a mutation in exon 3 of *CTNNB1 *[7]. Previous studies have revealed that epigenetic gene silencing of soluble Wnt antagonists, such as secreted Frizzled-related proteins (SFRPs), constitutes one of the major alterations resulting in constitutive activation of the canonical Wnt pathway in many tumors, particularly colorectal [8], bladder [9], gastric [10], and breast cancer [11]. In addition, abnormal CpG island methylation of key tumor suppressor genes has been reported in invasive cervical cancer [12]. We therefore examined whether promoter methylation of the genes encoding secreted Wnt antagonists is responsible for stabilization of β-catenin levels in cervical carcinoma.

Klotho has been characterized as systemic anti-aging hormone [13] and was originally identified in mice homozygous for the mutated allele (*kl^-/-^*). These mice show a human-like aging-related syndrome and develop multiple disorders such as hypogonadism, ectopic calcification, osteoporosis, skin atrophy, and pulmonary emphysema. Klotho-deficient mice die around 8-9 weeks of age [14]. In contrast, transgenic mice overexpressing Klotho have an extended life span that is 30% longer in males and 20% longer in females [15]. *KLOTHO *encodes a single-pass type-1 transmembrane protein as well as a truncated, secreted form derived from alternative RNA splicing. The major transcript in humans produces the secreted form, and the extracellular domain of the membrane-bound form is shed and then secreted. These soluble KLOTHO products function as circulating factors that are involved in multiple signaling pathways. Although the molecular mechanism of Klotho in the suppression of aging-related phenotypes is unknown, Klotho was shown to be a secreted antagonist of the Wnt signaling pathway [16]. Klotho inhibits Wnt signaling activity by forming a complex with Wnt3, which is a manner of the SFRP class of secreted Wnt antagonists. Recently, KLOTHO was identified as a potential tumor suppressor that inhibits the IGF-1 pathway and activates the FGF pathway in human breast cancer [17]. In this study, we hypothesized that *KLOTHO *encoding the secreted Wnt antagonist and that acts as a tumor suppressor may be a candidate target for epigenetic silencing in human cervical carcinoma.

## Results and Discussion

### Transcriptional repression of *KLOTHO *in human cervical cancer

**Figure 1:**
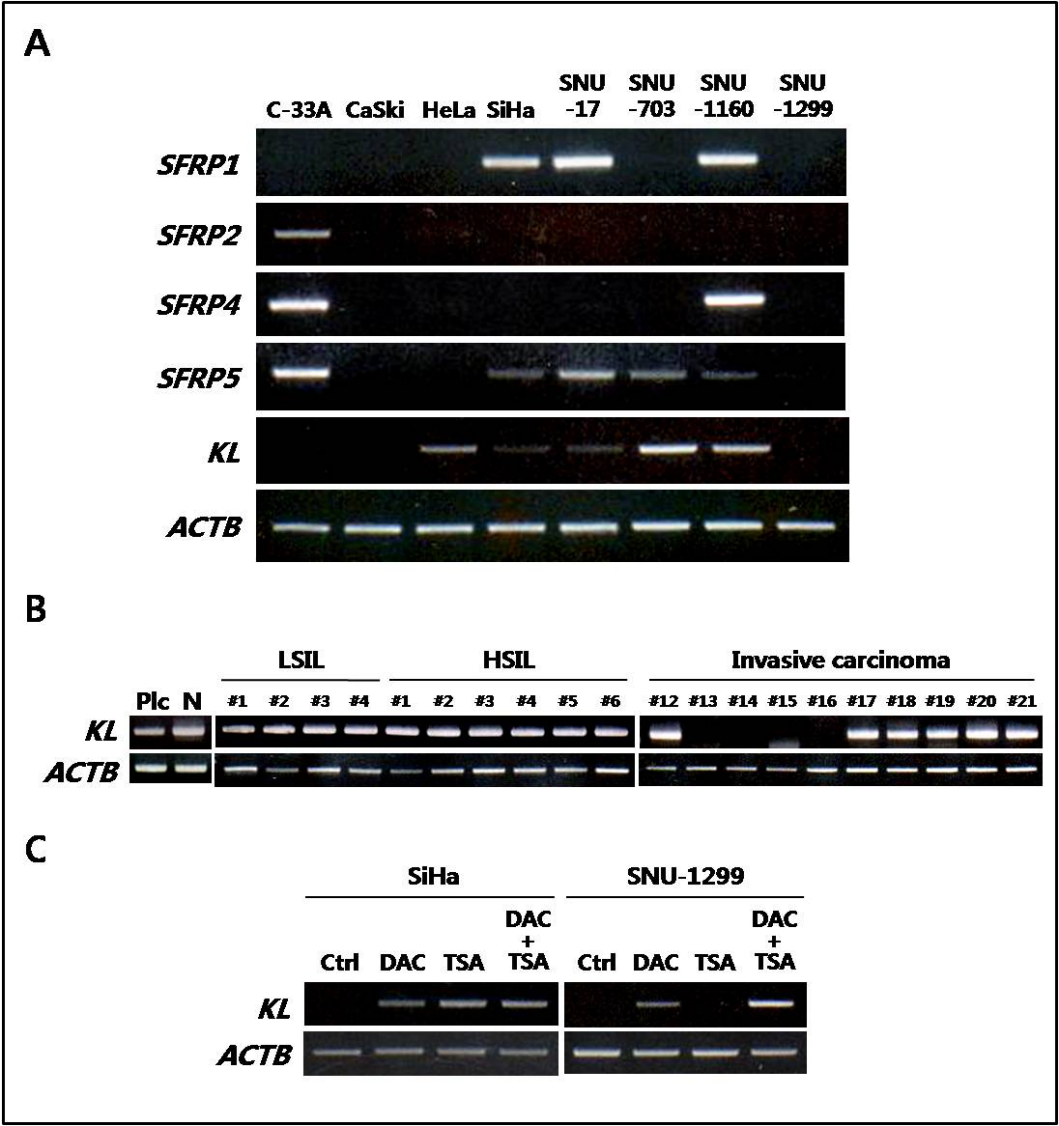
**Transcriptional repression of *KLOTHO *is associated with epigenetic inactivation in cervical cancer**. ***A***, RT-PCR analysis showing mRNA expression of the *secreted Frizzled-related protein (SFRP) *class of secreted Wnt antagonists in cervical cancer cell lines. Overall downregulation of *SFRPs *and variable mRNA levels of *KLOTHO *(*KL*) were detected. *ACTB *(encoding β-actin) is shown as an internal control. ***B***, The *KLOTHO *mRNA expression pattern in primary cervical tumor samples. cDNAs prepared from human placenta (Plc) and from normal (N) cervical tissue were used as positive controls for *KLOTHO *expression. ***C***, Transcriptional restoration of *KLOTHO *by either the DNMT inhibitor DAC or the HDAC inhibitor TSA in representative non-*KLOTHO*-expressing cell lines.

To investigate epigenetic gene silencing of the SFRP class of secreted Wnt antagonists in human cervical carcinoma, we first examined the mRNA levels of *SFRP1, SFRP2, SFRP4, SFRP5*, and *KLOTHO *in eight cervical cancer cell lines (Figure 1A). RT-PCR analysis showed complete loss of *SFRP *mRNAs in the CaSki, HeLa, and SNU-1299 cell lines and overall transcriptional downregulation of *SFRPs *in these cervical cancer cell lines. Complete transcriptional silencing of *SFRP1*, *SFRP2 *and *SFRP5 *was recently reported to be associated with promoter methylation in the CaSki and HeLa cell lines [18]. Although the SNU-703 and SNU-1160 cell lines expressed substantial levels of *KLOTHO *mRNA, loss of expression was found in the CaSki and SNU-1299 cell lines. In contrast with the SiHa and SNU-17 cell lines, which had extremely low levels of *KLOTHO *mRNA, the HeLa cell line showed a basal level of *KLOTHO *expression. To examine whether the transcriptional repression of *KLOTHO *also occurs in primary cervical tumors, we performed RT-PCR for *KLOTHO *in 4 samples of low-grade squamous intraepithelial lesion (LSIL), 6 samples of high-grade squamous intraepithelial lesion (HSIL), and 10 samples of invasive carcinoma (Figure 1B). It was previously reported that the expression of *KLOTHO *was predominant in the placenta, kidney, small intestine, and prostate [19]. cDNAs prepared from human placenta and normal cervical epithelial tissues were used as positive controls. Four of the ten invasive cervical carcinoma samples showed complete loss of *KLOTHO *expression, whereas maintenance of *KLOTHO *mRNA was observed in all of the early, preinvasive lesions (LSILs and HSILs). Thus, *KLOTHO *is downregulated in human cervical cancer cell lines and in invasive cervical carcinoma.

To determine whether the loss of *KLOTHO *expression is associated with epigenetic gene silencing, we used RT-PCR to examine transcriptional reactivation in representative cell lines lacking *KLOTHO *mRNA following treatment with either the DNA methyltransferase (DNMT) inhibitor 2'-deoxy-5-azacytidine (DAC) or the histone deacetylase (HDAC) inhibitor trichostatin A (TSA) (Figure 1C). DNA demethylation by DAC induced significant restoration of *KLOTHO *mRNA expression in the SNU-1299 cell line, but incomplete upregulation was seen in the SiHa cell line. When each cell line was treated with TSA alone, *KLOTHO *restoration was detected only in the SiHa cell line. A synergistic effect of DAC and TSA was observed in the SNU-1299 cell line, in which the *KLOTHO *mRNA was restored only with a DNA demethylating drug. Taken together, these data imply that the downregulation of *KLOTHO *in cervical cancer cell lines is correlated with epigenetic inactivation mechanisms involving DNA methylation and histone deacetylation.

### Promoter CpG hypermethylation of *KLOTHO *in cervical cancer

**Figure 2:**
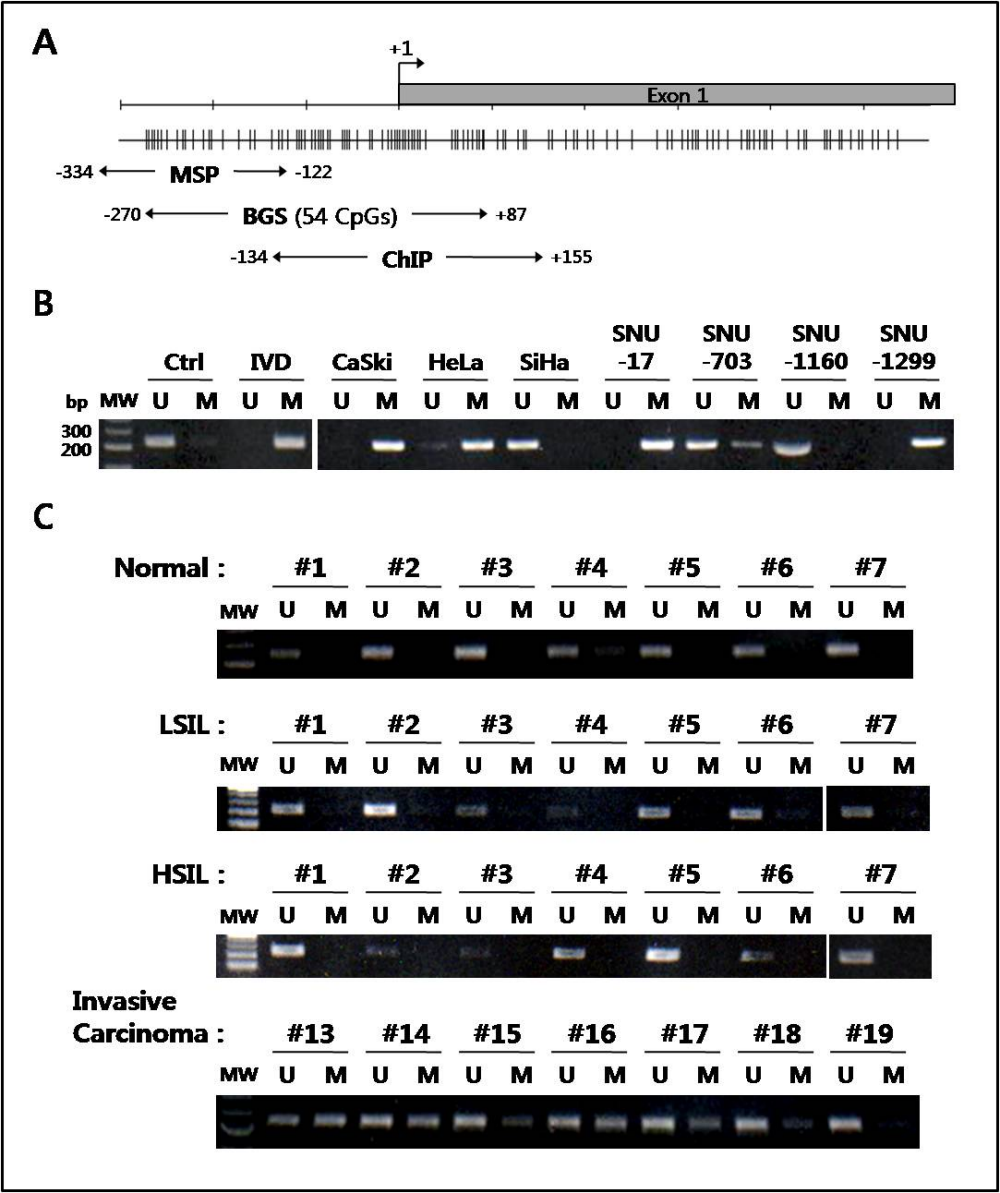
**Hypermethylation of the promoter region of *KLOTHO *in cervical cancer**. ***A***, Schematic representation of the upstream region of *KLOTHO *around the transcription start site (+1) and the primer location. The vertical lines represent the CpG dinucleotides. ***B***, MSP assay of *KLOTHO *in cervical cancer cell lines. *In vitro *methylated DNA (IVD) from a placenta was used as a positive control for methylated DNA. ***C***, Representative results of the MSP analysis of *KLOTHO *in human cervical tissue samples. Normal, LSIL, HSIL, and invasive carcinoma represent the histopathologic grades of cervical carcinogenesis.

**Figure 3:**
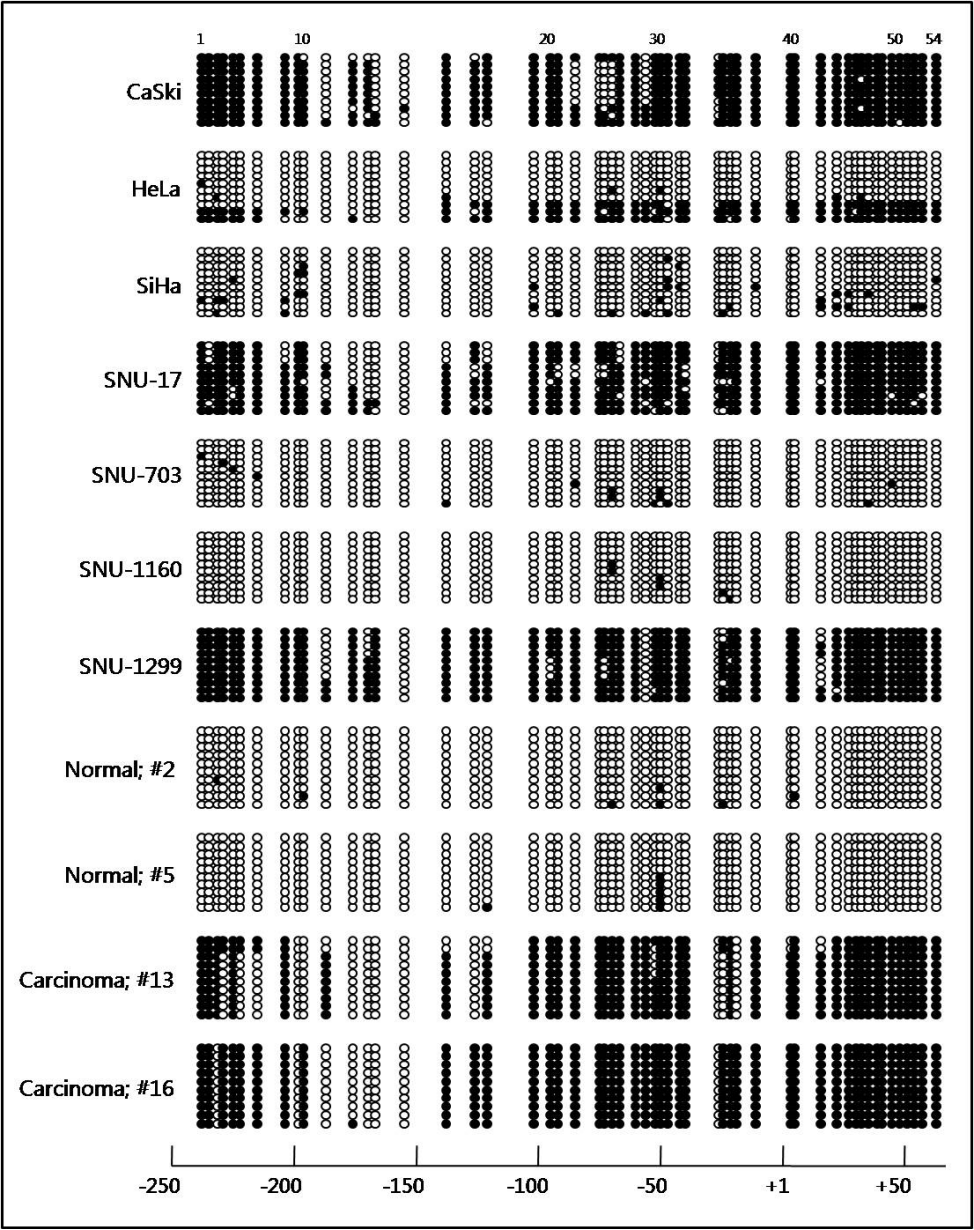
**Bisulfite genomic sequencing analysis of the *KLOTHO *promoter region in cervical cancer**. Each row of circles represents the DNA sequence of individual clones. The open and filled circles represent the unmethylated and methylated CpG sites, respectively. Partial methylation in the HeLa cell line was detected.

To analyze the methylation status of the CpG islands in the *KLOTHO *promoter region, methylation-specific PCR (MSP) and bisulfite genomic sequencing (BGS) analysis were performed in cervical cancer cell lines. As shown in Figure 2A, a dense GC-rich region was identified around the transcription start site of *KLOTHO*. Positive controls for unmethylated alleles were verified with MSP using genomic DNA from human placenta. Positive controls for methylated alleles were confirmed by MSP of *in vitro *methylated DNA (IVD) of the same material. Unmethylated (U) products were predominant in the promoter region of control DNA from human placenta; however, methylated (M) products were exclusively amplified from an IVD template (Figure 2B). The promoter methylation profile obtained from the MSP analysis of *KLOTHO *represented a complete methylation pattern in the CaSki, SNU-17, and SNU-1299 cell lines. In contrast, the SNU-703 and SNU-1160 cell lines showed an unmethylated *KLOTHO *promoter region. These results were consistent with the RT-PCR analysis: the *KLOTHO*-expressing cell lines SNU-703 and SNU-1160 exhibited unmethylated promoter CpGs, and the non-*KLOTHO*-expressing cell lines CaSki, SNU-17, and SNU-1299 showed methylated CpGs. To further determine the methylation density of the *KLOTHO *promoter region, which encompasses 54 CpGs, we performed a BGS analysis of the cervical cancer cell lines (Figure 3). Although extensive hypermethylation of the *KLOTHO *promoter region was prominent in the cell lines that lacked *KLOTHO *expression (CaSki, SNU-17, and SNU-1299), the *KLOTHO*-expressing cell lines (SNU-703 and SNU-1160) tended to have completely unmethylated CpG islands. These data strongly suggest that transcriptional repression of *KLOTHO *is associated with aberrant promoter hypermethylation in cervical cancer cell lines.

We next examined the methylation status of primary cervical tumor samples to investigate whether *KLOTHO *undergoes abnormal methylation in association with the histopathologic grades of squamous intraepithelial lesions (Figure 2C). Promoter methylation was not detected in the normal cervical samples (n = 7), the LSIL (n = 30) or the HSIL (n = 37), but extensive methylation was observed exclusively in nine of 22 (41%) of the invasive carcinoma cases. Based on these observations, the number of non-*KLOTHO*-expressing tissue samples (Figure 1B) may be consistent with the methylation frequency of primary cervical tumors. To confirm the MSP results, we carried out bisulfite sequencing analysis using representative cases, including two normal and two invasive carcinoma samples (Figure 3). The overall methylation of the *KLOTHO *promoter region in the invasive carcinoma cases was in good agreement with the MSP data, whereas a fully unmethylated pattern was observed in the normal samples. Thus, epigenetic silencing of *KLOTHO *due to promoter hypermethylation may occur in an invasive carcinoma phase-specific manner during cervical carcinogenesis.

### Histone deacetylation is the major epigenetic silencing mechanism for *KLOTHO *in the SiHa cell line

**Figure 4:**
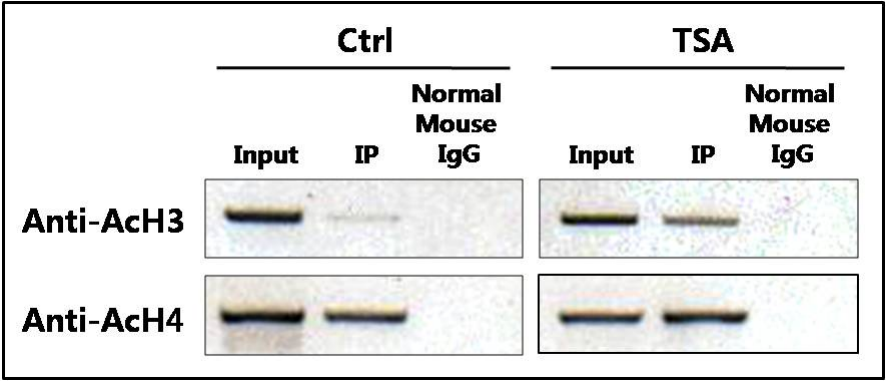
**ChIP analysis of the *KLOTHO *promoter region in SiHa cells**. Antibodies against acetylated histone H3 (AcH3) and H4 (AcH4) were used to isolate acetylated chromatin fragments from lysates of control and TSA-treated SiHa cells, respectively. DNA fragments corresponding to the *KLOTHO *promoter around the transcription start site were amplified by PCR.

A chromatin immunoprecipitation (ChIP) assay using anti-acetyl histone H3 (AcH3) and H4 (AcH4) antibodies was performed to determine whether SiHa cells utilize local histone modification as a mechanism of *KLOTHO *silencing. The immunoprecipitated DNA was analyzed by PCR to elucidate the histone H3 and H4 acetylation level of the *KLOTHO *promoter region. Figure 2A shows the promoter region around the transcription start site (+1) that was analyzed by the ChIP assay. As shown in Figure 4, marked differences were observed between the acetylation level of histones H3 and H4 in untreated SiHa cells. Although very low levels of PCR products were detected in the control SiHa cells, TSA treatment resulted in a dramatic amplification of specific DNA fragments immunoprecipitated with the AcH3 antibody. This result suggested that the *KLOTHO *promoter is enriched in deacetylated histone H3, leading to repressive histone modification in the SiHa cell line. On the other hand, the acetylation level of histone H4 seemed to not be associated with restoration of *KLOTHO *mRNA after treatment with TSA in the SiHa cell line. Suzuki et al. reported that a group of genes lacking promoter CpG methylation tended to be reactivated after HDAC inhibition alone [20]. Our study identified unmethylated CpGs present in the *KLOTHO *promoter in the SiHa cell line (Figure 3). Thus, deacetylated histone H3 in the promoter region is strongly correlated with epigenetic silencing of *KLOTHO *in the SiHa cell line.

### KLOTHO functions as a secreted Wnt antagonist in cervical cancer

**Figure 5:**
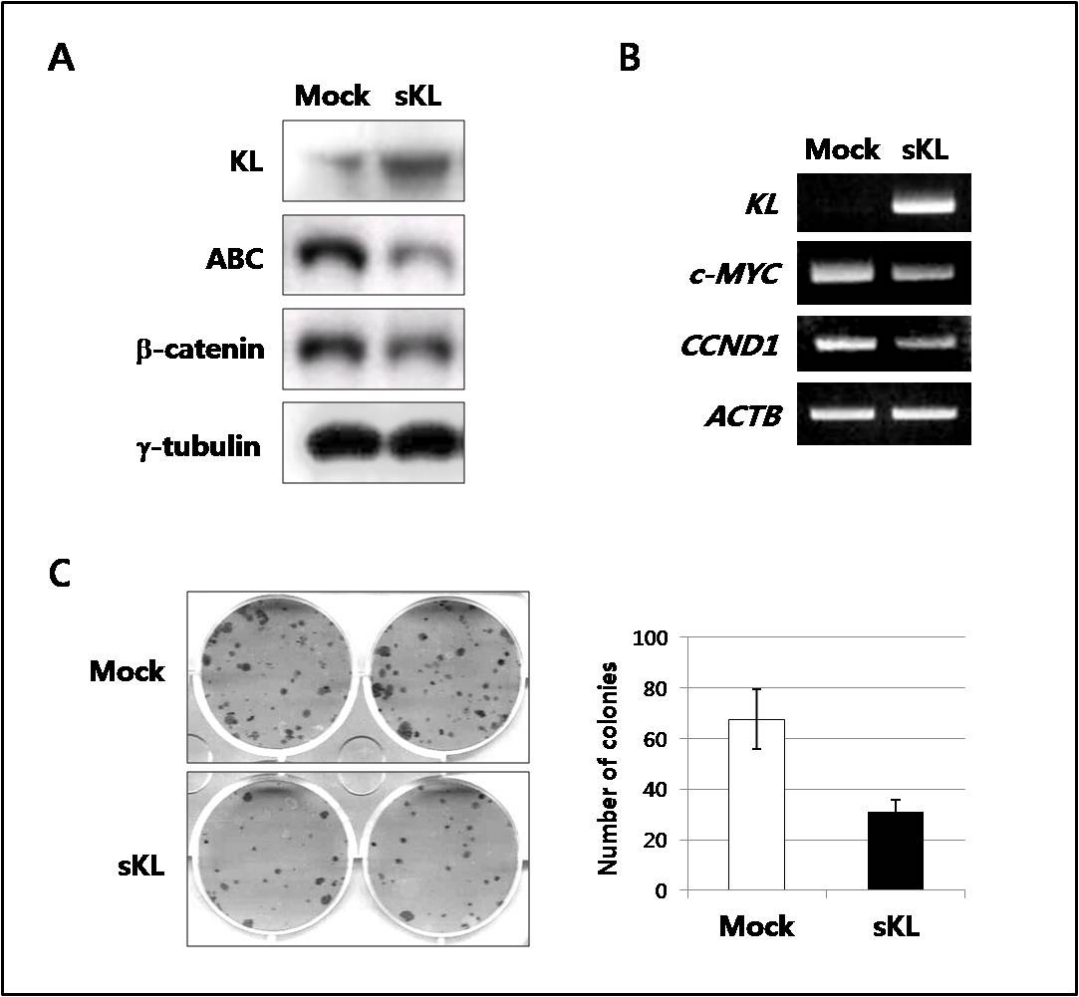
**Inhibition of the Wnt/β-catenin pathway and colony growth following ectopic expression of the secreted form of KLOTHO (sKL) in a cervical cancer cell line**. ***A***, Immunoblot analysis of KLOTHO, total β-catenin, and active β-catenin (ABC) in CaSki cells transfected with either the sKL expression vector or the empty vector. ***B***, RT-PCR analysis of TCF/β-catenin target genes, i.e., *c-MYC *and *CCND1*, in CaSki cells transfected with either the sKL expression vector or the empty vector. *ACTB *is shown as an internal control. ***C***, Colony formation assay showing that restoration of KLOTHO suppresses tumor cell growth in the CaSki cell line.

To assess whether KLOTHO inhibits the Wnt signaling pathway in cervical cancer, the CaSki cell line was transfected with a cDNA expression vector that produces a secreted form of KLOTHO (pcDNA3.1/V5-His-sKL). Ectopic expression of sKL protein resulted in a decrease in total β-catenin levels and a dramatic reduction in the active form of β-catenin (ABC), which is dephosphorylated on S37 or T41 residues (Figure 5A). The expression of representative T-cell factor (TCF)/β-catenin target genes, including *c-MYC *and *CCND1 *(encoding Cyclin D1), was markedly decreased in the CaSki cell line when the overexpression of sKL was induced (Figure 5B). c-*MYC *is a specific oncogene that is commonly activated during cervical carcinogenesis [21]. These findings indicate that the canonical Wnt pathway is inhibited by a secreted form of KLOTHO in a cervical cancer cell line. Finally, we performed a colony formation assay to test the tumor-suppressor activity of KLOTHO as a result of inhibition of Wnt/β-catenin signaling. As shown in Figure 5C, CaSki cells transfected with the sKL expression vector showed 57% fewer colonies compared to the empty vector. This result shows that KLOTHO functions as a potential tumor suppressor and that loss of *KLOTHO *may contribute to cervical carcinogenesis.

Abnormal activation of the canonical Wnt pathway due to epigenetic deregulation of Wnt antagonists is thought to play a crucial role during multi-step cervical tumorigenesis. In addition, several extracellular antagonists of the Wnt pathway, including *SFRP1*, *SFRP2 *[18], *DKK1 *[22], and *DKK3 *[23], were reported to be downregulated by methylation in cervical cancer. Baylin et al. proposed that epigenetic changes may cause cancer cells to become "addicted" to altered signaling pathways, e.g., the Wnt/β-catenin pathway, during the early stages of tumorigenesis [24]. These early epigenetic events that occur in the secreted Wnt-antagonist gene family may allow these cells, i.e., HPV-infected cervical epithelial cells, to acquire genetic or epigenetic alterations in the same pathway, providing the cell with selective advantages that promote tumor development. In our unpublished studies, frequent epigenetic inactivation of the *SFRP *gene family was observed during the early, preinvasive phase of cervical carcinogenesis preceding *KLOTHO *silencing. Our results suggest that loss of *KLOTHO *during the late phase may be another important step toward invasion of cervical cancer cells with aberrant Wnt signaling. Our current study shows that *KLOTHO *is frequently downregulated both in cervical cancer cell lines and in invasive carcinomas of the primary cervical tumors and that transcriptional repression of *KLOTHO *is associated with promoter hypermethylation. We show here that epigenetic aberrations of *KLOTHO *occurred in an invasive carcinoma phase-specific manner and that a loss of function of KLOTHO induced oncogenic activation of TCF/β-catenin target genes in a cervical carcinoma. Thus, epigenetic aberrations of the secreted Wnt antagonist KLOTHO may contribute to the development of cervical cancer. Our analysis is the first to establish that *KLOTHO*, the gene for a secreted Wnt-antagonist undergoes epigenetic silencing in human cancers. It remains to be determined whether *KLOTHO *is epigenetically inactivated in other types of human cancer. Moreover, *KLOTHO *may be a potential target for the treatment of invasive carcinomas and as a prognostic biomarker of cervical cancer.

## Conclusions

This study suggests that transcriptional repression of *KLOTHO *is correlated with promoter CpG hypermethylation in cervical cancer. Frequent (41%) promoter methylation of *KLOTHO *occurred in invasive carcinoma but not in normal cervical tissues or during the early, preinvasive phase of primary cervical tumors. Our study also showed that histone deacetylation was responsible for silencing of *KLOTHO *in the SiHa cell line. Functional loss of KLOTHO due to epigenetic silencing may contribute to aberrant activation of the canonical Wnt pathway in cervical carcinoma.

## Methods

### Cell culture and drug treatment

The human cervical cancer cell line C-33A was obtained from the American Type Culture Collection (Manassas, VA), and CaSki, HeLa, SiHa, SNU-17, SNU-703, SNU-1160, and SNU-1299 were obtained from the Korean Cell Line Bank (Seoul, Korea) [25]. The C-33A and CaSki cell lines were derived from carcinomas, SiHa and all the SNU-series cell lines were derived from squamous cell carcinomas, and the HeLa cell line was derived from an adenocarcinoma. The HPV-negative cell line C-33A, the HPV-16-positive cell line SiHa, and the HPV-18-positive cell line HeLa were grown in Dulbecco's Modified Eagle Medium (Invitrogen) supplemented with 10% fetal bovine serum, 100 U/ml penicillin, 100 μg/ml streptomycin, and 250 ng/ml amphotericin B. The HPV-16-positive cell lines CaSki, SNU-17, SNU-703, and SNU-1299, and the HPV-18-positive cell line SNU-1160 were grown in RPMI-1640 medium (Invitrogen) supplemented with 10% fetal bovine serum, 100 U/ml penicillin, 100 μg/ml streptomycin, and 250 ng/ml amphotericin B. All cells were maintained at 37°C in a humidified incubator with 5% CO_2_. We treated the cervical cancer cell lines with DAC (Sigma) for 72 h and TSA (Sigma) for 24 h.

### Tissue samples and nucleic acid preparation

The uterine cervical tissues were obtained either by punch biopsy or hysterectomy from patients with cervical intraepithelial neoplasm. The tissues were fixed in 10% neutral buffered formalin for 6 to 12 h, and then embedded in paraffin. The histopathologic diagnoses of tissues were made by a pathologist. All patients provided informed consent, and the study was approved by the institutional review board of Soonchunhyang University Cheonan Hospital. After identification of the uterine cervical epithelial lesions on hematoxylin and eosin-stained slides, a pathologist manually dissected the epithelial portions with a 26 gauge needle using a light microscope and added the cells to lysis buffer (10 mM Tris-HCl, pH 8.0; 0.1 mM EDTA, pH 8.0; 2% SDS; 0.15 mg proteinase K). Samples were incubated at 60°C until they were completely lysed, and then genomic DNA was extracted with phenol:chloroform:isoamyl alcohol (25:24:1) and ethanol precipitated at -20°C overnight. The DNA pellet was then dissolved and quantified using a NanoDrop^® ^ND-1000 Spectrophotometer (Nanodrop Technologies, USA). After the same tissue samples were treated with lysis buffer at 55°C for 3 h, total RNA was extracted using TRIzol Reagent (Invitrogen) according to the manufacturer's protocol.

### Reverse transcription-PCR

Total RNA was isolated from cell lines using an RNeasy Mini kit (Qiagen) following the manufacturer's instructions, and cDNA synthesis was performed with 1 μg of the total RNA using the ImProm-II™ Reverse Transcription System (Promega). The primer sequences for RT-PCR of the *KLOTHO *cDNA were 5'-ACTCCCCCAGTCAGGTGGCGGTA-3' (forward) and 5'-TGGGCCCGGGAAACCATTGCTGTC-3' (reverse). All amplifications were carried out with AccuPower PCR PreMix (Bioneer). Human *ACTB *(encoding β-actin) was amplified as an endogenous control.

### Methylation analysis

Genomic DNA was extracted from cell lines using a DNeasy Tissue kit (Qiagen), and bisulfite modification of genomic DNA was performed using an EZ DNA Methylation kit (Zymo Research), following the manufacturers' instructions. Bisulfite-converted genomic DNA was amplified using specific MSP primer sets that discriminate between unmethylated (U) and methylated (M) promoter region of *KLOTHO*. The sequences of the U-specific primer set were 5'-AGAGGATGTGTGGTAGGTAAAGAG-3' (forward) and 5'-ACAAACCAAAACTACCTCCACCCT-3' (reverse). The sequences of the M-specific primer set for the M1 region were 5'-AGAGGACGCGCGGTAGGTAA-3' (forward) and 5'-ACGAACCGAAACTACCTCCGC-3' (reverse). We treated the human placental DNA with SssI methylase (New England Biolabs) following the manufacturer's protocol for use as positive control for methylation analysis. Bisulfite genomic sequencing was performed to determine the methylation density of the 357 bp fragment containing 54 CpGs at the 5'-end region of *KLOTHO*. The primer sequences were 5'-GGGAGTTGGGAGAAATAGGTGT-3' (forward) and 5'-CCAAACCCAACAACACCAACAAC-3' (reverse). PCR products were purified and subcloned into the pGEM-T Easy Vector (Promega) for subsequent sequencing analysis.

### Chromatin immunoprecipitation analysis

ChIP analysis was carried out using an EZ ChIP kit (Millipore) following the manufacturer's protocol. SiHa cells (4 × 10^5 ^cells) were plated in a 100-mm culture dish and incubated for 24 h. Formaldehyde-treated cells were resuspended in SDS lysis buffer, and the cell lysates were sheared by sonication. The chromatin fragments were immunoprecipitated with antibodies against acetylated histone H3 (Millipore) or H4 (Millipore), and then the purified DNA was analyzed by PCR using the primer pairs: 5'-AGTCCCGGCTCGCAGGTAATTATTG-3' (forward) and 5'-AGGAGGCCGCGAGAAACGGG-3' (reverse), which were designed to amplify nucleotides -134 to +155 of *KLOTHO*.

### Transfection, immunoblotting, and colony formation assay

The secreted form of human *KLOTHO *(sKL) cDNA cloned into pcDNA3.1/V5-His expression vector (Invitrogen) was a generous gift from Michael J. Econs (Indiana University School of Medicine, Indianapolis, IN, USA). CaSki cells were plated at 1 × 10^6 ^cells/60- mm culture dish 24 h prior to transfection and were transfected with 5 μg of either the sKL expression vector or an empty control vector for 5 h in serum-free medium using Lipofectamine 2000 (Invitrogen). After replacing the DNA-Lipofectamine complex-containing medium with complete growth medium, transfected cells were incubated for 72 h. For immunoblotting, 20 μg of protein per lane were separated on polyacrylamide gels and blotted onto PVDF membranes (Millipore). Antibodies against KLOTHO (Santa Cruz), total β-catenin (Cell Signaling), and active β-catenin (Millipore) were used to probe the blot. For the colony formation assay, CaSki cells were plated in 6-well plates after transfection and selected with 1 mg/ml G418 (Invitrogen) for 17 d. Subsequently, colonies were stained with crystal violet, and duplicate wells were counted.

## Competing interests

The authors declare that they have no competing interests.

## Authors' contributions

JL conceived the studies, acquired the majority of the epigenetic analysis data and wrote the manuscript. DJ and CK provided the tissue samples and participated in some of the experiments. JK performed the immunoblotting and colony formation assay. SL, JP, BC, SJ and LY participated in some of the experiments. YH, YY, KIK and JL contributed to the concept of the study. IY contributed to the concept of the study and helped draft the manuscript. SJ and DHB provided the patient samples. ML oversaw the experimental work, participated in its design and coordination and helped draft the manuscript. All authors have read and approved the final manuscript.
